# Supplementary material for: Degradable and Non-Degradable Chondroitin Sulfate Particles with the Controlled Antibiotic Release for Bacterial Infections
Source: Pharmaceutics. 2022 Aug 20;14(8):1739. doi: 10.3390/pharmaceutics14081739 (PMC9415033; doi:10.3390/pharmaceutics14081739)
Supplement: Supplementary file 1 [file pharmaceutics-14-01739-s001.zip › pharmaceutics-1856750-supplementary.pdf]

Degradable and Non-Degradable Chondroitin Sulfate Particles with the Controlled Antibiotic Release for Bacterial Infections

Selin S. Suner [1], Mehtap Sahiner [2], Ramesh S. Ayyala [3] and Nurettin Sahiner [1,3,4,*]

[1] Department of Chemistry & Nanoscience and Technology Research and Application Center, Canakkale Onsekiz Mart University Terzioglu Campus, Canakkale 17100, Turkey
[2] Bioengineering Department, Engineering Faculty, Canakkale Onsekiz Mart University Terzioglu Campus, Canakkale 17100, Turkey
[3] Department of Ophthalmology, Morsani College of Medicine, University of South Florida, Tampa, FL 33612, USA
[4] Department of Chemical and Biomolecular Engineering, University of South Florida, Tampa, FL 33620, USA
* Correspondence: sahiner71@gmail.com or nsahiner@usf.edu

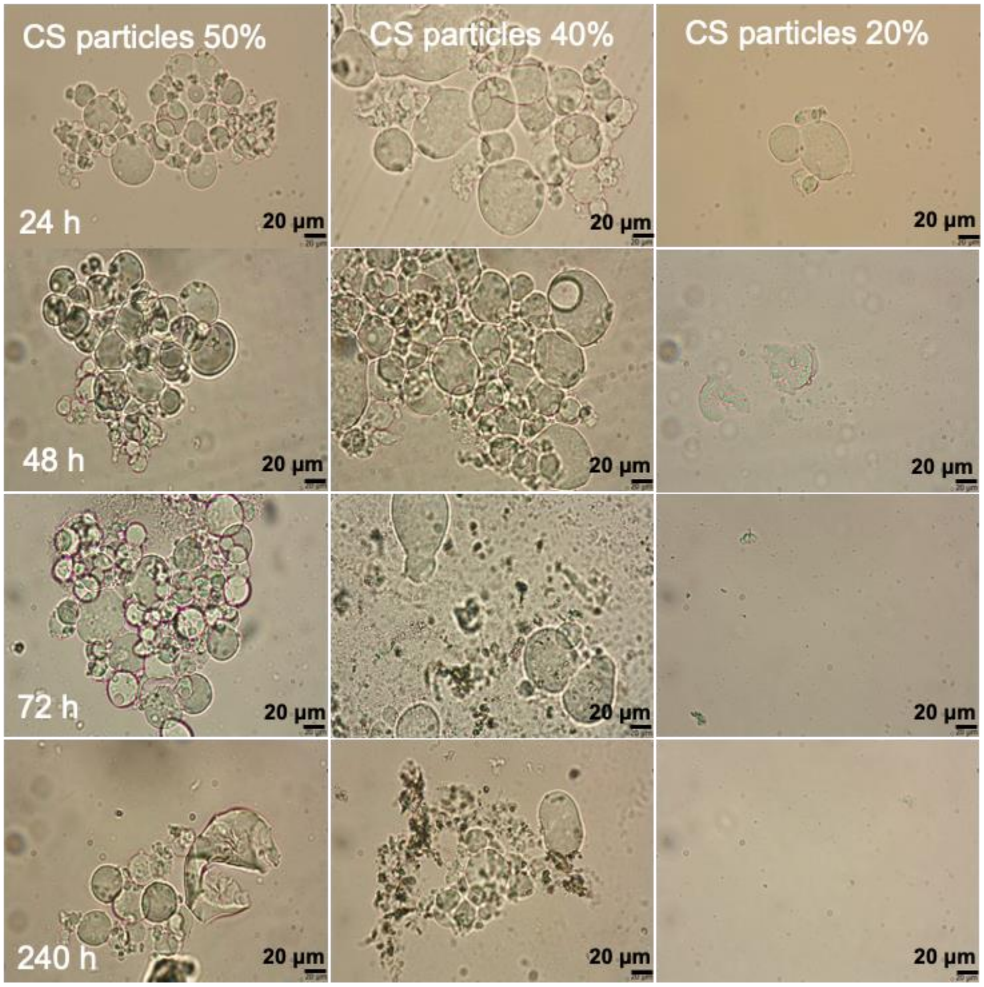

Figure S1. Optical microscope images of CS particles crosslinked at 50, 40, and 20% mole ratio of CS repeating unit incubated for 24 h, 48 h, 72 h, and 240 h in physiological conditions at pH 7.4 and 37 ℃.

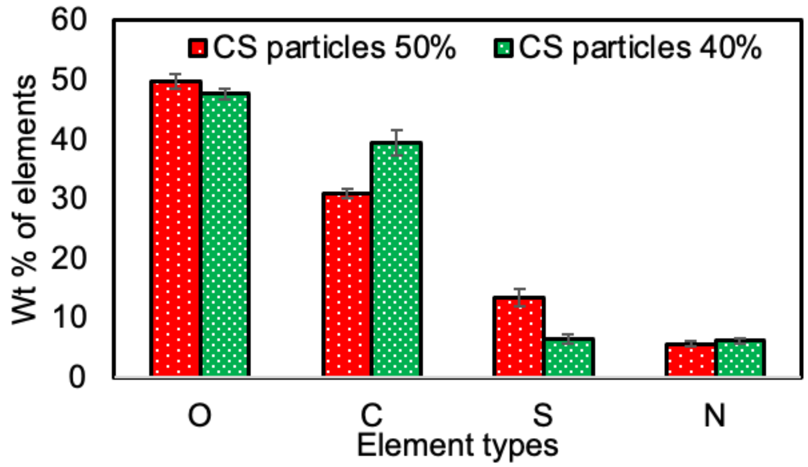

Figure S2. Elemental analysis of the CS particles crosslinked at 50%, and 40%.
